# Viability, Apoptosis, Proliferation, Activation, and Cytokine Secretion of Human Keratoconus Keratocytes after Cross-Linking

**DOI:** 10.1155/2015/254237

**Published:** 2015-01-28

**Authors:** Xuefei Song, Tanja Stachon, Jiong Wang, Achim Langenbucher, Berthold Seitz, Nóra Szentmáry

**Affiliations:** ^1^Department of Ophthalmology, Saarland University Medical Center, Kirrberger Straße 100, 66424 Homburg, Germany; ^2^Department of Ophthalmology, The First Affiliated Hospital of Zhengzhou University, Zhengzhou 450052, China; ^3^Experimental Ophthalmology, Saarland University, 66424 Homburg, Germany

## Abstract

*Purpose*. The purpose of this study was to determine the impact of cross-linking (CXL) on viability, apoptosis, proliferation, activation, and cytokine secretion of human keratoconus (KC) keratocytes, *in vitro*. *Methods*. Primary KC keratocytes were cultured in DMEM/Ham's F12 medium supplemented with 10% FCS and underwent UVA illumination (370 nm, 2 J/cm^2^) during exposure to 0.1% riboflavin and 20% Dextran in PBS. Twenty-four hours after CXL, viability was assessed using Alamar blue assay; apoptosis using APO-DIRECT Kit; proliferation using ELISA-BrdU kit; and CD34 and alpha-smooth muscle actin (*α*-SMA) expression using flow cytometry. Five and 24 hours after CXL, FGFb, HGF, TGF*β*1, VEGF, KGF, IL-1*β*, IL-6, and IL-8 secretion was measured using enzyme-linked-immunoabsorbent assay (ELISA). *Results*. Following CXL, cell viability and proliferation decreased (*P* < 0.05; *P* = 0.009), the percentage of apoptotic keratocytes increased (*P* < 0.05) significantly, and CD34 and *α*-SMA expression remained unchanged (*P* > 0.06). Five hours after CXL, FGFb secretion increased significantly (*P* = 0.037); however no other cytokine secretion differed significantly from controls after 5 or 24 hours (*P* > 0.12). *Conclusions*. Cross-linking decreases viability, triggers apoptosis, and inhibits proliferation, without an impact on multipotent hematopoietic stem cell transformation and myofibroblastic transformation of KC keratocytes. CXL triggers FGFb secretion of KC keratocytes transiently (5 hours), normalizing after 24 hours.

## 1. Introduction

Keratoconus (KC) is the most common primary ectasia, characterized by localized corneal thinning leading to protrusion of the thinned cornea. It usually occurs in the second decade of life and affects both genders and all ethnicities [1, 2]. The estimated prevalence in the general population is 54 per 100,000 [3].

In recent years the technique of corneal collagen cross-linking (CXL), which has the ability to stop the progression of corneal ectasia in KC, has been developed [4, 5].

Infectious keratitis is a potentially blinding ocular disease of the cornea. Besides antimicrobial drugs, the use of CXL as photodynamic therapy (PDT) is also considered as potential alternative in the management of infectious keratitis [6–10]. Thus, CXL has also been described as riboflavin-UVA-photodynamic-inactivation (riboflavin-UVA-PDI) [11].

Recent studies have shown that, in normal keratocytes, CXL decreases viability and induces myofibroblastic transformation and multipotent haematopoietic stem cell transformation but has no impact on apoptosis of the cells [12, 13]. In the short term, the iatrogenic thinning or swelling effect of the riboflavin solution on the cornea during CXL could also be described, depending on the properties of the riboflavin solution used [14]. However, the impact of CXL on human KC keratocytes has not yet been analyzed.

Growth factors and interleukins regulate proliferation, motility, cellular interactions, differentiation, apoptosis, and other functions that occur during wound healing or inflammation in the cornea [15].

Regarding growth factor and interleukin secretion, we recently determined in normalkeratocytes that CXL decreases FGFb, VEGF, and HGF secretion 5 hours and FGFb and KGF secretion 24 hours after treatment and increases IL-6 secretion after 24 hours. In the short term, CXL did not have an impact on TGF*β*1 and IL-8 secretion of normal keratocytes [16, 17].

Observing the differences between normal and KC keratocytes in myofibroblastic transformation, multipotent hemopoietic stem cell transformation, and apoptosis following PDT, we expected a difference in growth factor and interleukin secretion of KC keratocytes after CXL, compared to normals. In addition, several authors reported on the occurrence of early bacterial or acanthamoeba keratitis following CXL therapy [3, 16, 17]. To the best of our knowledge, the secretion of growth factors and interleukins in human KC keratocytes has not yet been analyzed, though these cytokines may play an important role in postoperative corneal wound healing of KC patients following CXL.

The purpose of our study was to determine the impact of CXL on viability, apoptosis, proliferation, activation, and growth factor and interleukin secretion of human KC keratocytes,* in vitro*.

## 2. Materials and Methods

In accordance with the Declaration of Helsinki, the ethics committee of the University of Saarland approved our study. Written informed consent was obtained by all participants in this study. The ethics committee of the University of Saarland approved this consent procedure.

### 2.1. Materials

Dulbecco's Modified Eagle Medium: nutrient mixture F-12 (DMEM/F12); fetal bovine serum (10%); P/S (1% of 10,000 U/mL penicillin and 10 mg/mL streptomycin); and 0.05% trypsin/0.02% ethylenediaminetetraacetic acid (EDTA) were purchased from PPA Laboratories (Pasching, Austria) and Alamar blue was from Invitrogen (Karlsruhe, Germany). Collagenase A, Dispase II, and Cell Proliferation ELISA-BrdU (colorimetric) were obtained from Roche Diagnostics (Mannheim, Germany). The APO-DIRECT Kit and all tissue culture plastics were sourced from BD Biosciences (Heidelberg, Germany). Mouse Anti-Human CD34-FITC was from Biozol (Eching, Germany) and anti-alpha smooth muscle actin antibody (FITC) was obtained from Abcam (Cambridge, USA). Riboflavin-5-phosphate and Dextran were purchased from Sigma-Aldrich (Heidelberg, Germany).

### 2.2. Isolation of Primary Human Keratoconus Keratocytes

Keratocytes were isolated from the explanted corneal buttons of 5 KC patients who underwent penetrating keratoplasty at the Department of Ophthalmology of Saarland University Medical Center, Homburg/Saar, Germany. KC keratocytes were isolated as described previously [18–20]. In summary, the human corneoscleral buttons were aseptically rinsed in phosphate-buffered saline (PBS) (PAA, Pasching, Austria) before removal of the endothelium including the Descemet's membrane using a sterile surgical disposable scalpel. The corneal button was incubated in DMEM/F12 (PAA, Pasching, Austria), containing 2.4 U/mL Dispase II (Roche Diagnostics, Mannheim, Germany) for 4 hours at 37°C. Afterwards, the corneal button was washed with PBS several times and the corneal epithelium was removed with surgical disposable scalpel. The remaining corneal stroma was digested in DMEM/F12 with 1.0 mg/mL collagenase A (Roche Diagnostics, Mannheim, Germany) for 8–10 hours at 37°C. The digested tissue and cells were pipetted three times and centrifuged at 800 g for 7 minutes and finally resuspended in 1.0 mL culture medium, which consisted of basic medium (DMEM/F12) supplemented with 10% fetal bovine serum (FBS) (Life Technologies, Darmstadt, Germany) and 1% P/S (1% of 10000 U/mL penicillin and 10 mg/mL streptomycin) (PAA, Pasching, Austria). The cell suspension and small tissue pieces were seeded in 6-well plates and the medium was changed twenty-four hours after seeding. The medium was changed every 2 to 3 days until the keratocytes reached confluence. The cells were subcultured in 25 cm^2^ culture flasks after 5 to 10 days following dispersal with 0.05% trypsin-EDTA (0.05% trypsin/0.02% ethylenediaminetetraacetic acid) (PAA, Pasching, Austria) for 3 to 5 minutes and the passages 4 to 8 of the cells were used for experiments.

### 2.3. CXL before Viability, Apoptosis, Proliferation, and CD34 and Alpha-SMA Expression Measurements

Human KC keratocytes were seeded in tissue culture plates and were allowed to grow for 48 hours before photodynamic treatment. For viability, apoptosis, proliferation, and CD34 and alpha-SMA expression measurements, 0.5% and 0.1% concentrations of riboflavin-5-phosphate were diluted in 20% Dextran-PBS. Cells were washed with PBS once before the above riboflavin solution was added. Then, the cells were exposed directly to UVA-light (370 nm) for 4 minutes 10 seconds with an irradiation dose of 8 mW/cm^2^ (2 J/cm^2^). Following illumination, the cells were washed twice with PBS, fed with culture medium, and cultivated at 37°C for twenty-four hours before measurements.

### 2.4. Determination of Viability (Phototoxicity)

Cell viability was evaluated using the Alamar blue assay as follows: Human KC keratocytes were seeded in twenty-four-well cell culture plates at a density of 7.5 × 10^3^ cells/cm^2^. At twenty-four hours after illumination, the Alamar blue solution was diluted with culture medium to a final concentration of 10% and 500 *μ*L of this solution was added to each well. As a negative control, Alamar blue solution was added to a well without cells. Thereafter, all plates were exposed to an excitation wavelength of 560 nm, and the emission at 616 nm was recorded using a 96-well microplate reader (WALLAC 1420 Multilabel Counter, Perkin Elmer, Life Sciences, Wellesley, MA, USA).

### 2.5. Determination of Apoptosis

To determine the relative number of apoptotic KC keratocytes using the APO-DIRECT kit, the cells were seeded in 6-well cell culture plates at a density of  7.5 × 10^3^ cells/cm^2^ and underwent CXL as described above. The cells were then harvested twenty-four hours after the CXL. First, the culture medium was discarded and the cells were trypsinized before centrifugation. Then, the cells were suspended in 1% paraformaldehyde at a density of 1.0 × 10^6^ cells/mL and placed on ice for 30–60 minutes. Afterwards, the cells were washed twice with PBS and stored for 30 minutes at −20°C following the addition of 1 mL ice cold 70% ethanol. After removing the ethanol carefully by aspiration, fixed cells were resuspended twice in 1.0 mL wash-buffer. The control cells and the probes were resuspended in 50 *μ*L DNA-labeling-solution (FITC marked dUTP) and the cells were washed twice before resuspending the cell pellet in 500 *μ*L PI/RNase staining buffer. The cells were incubated in the dark for at least 30 minutes at room temperature prior to analysis using a FACSCanto flow cytometer (BD Biosciences, Heidelberg, Germany).

### 2.6. Determination of Cell Proliferation

To detect the impact of CXL on the proliferation rate of KC keratocytes, the proliferation was determined with the cell proliferation ELISA-BrdU kit which allows the measurement of BrdU incorporation into newly synthesized cellular DNA. We performed the CXL procedure as described before [18] and tested the impact of CXL on the proliferation rate of KC keratocytes at 1 h before and 24 h after CXL using 0.05% and 0.1% riboflavin concentrations. Keratocytes were seeded in a 96-multiwell plate at a density of 2 × 10^3^ cells/well in 100 *μ*L of culture medium. The test was performed according to the manufacturer's protocol. BrdU was added to the keratocytes at the tissue plates and incubated at 37°C for 4 h (BrdU incorporation). After removing the culture medium, the cells were fixed with FixDenat (provided with the test kit) followed by incubation with anti-BrdU-POD (monoclonal antibody to the thymidine-analogue 5-bromo-2′-deoxyuridine Fab fragments with peroxidase conjugated), which binds the incorporated DNA. After adding the substrate solution, the immune complexes were detected using an ELISA reader, Model 550 (Bio-Rad Laboratories GmbH, München, Germany).

### 2.7. Determination of CD34 and Alpha-SMA Expression

KC keratocytes were seeded in 6-well cell culture plates at a density of 4.0 × 10^3^ cells/cm^2^ and underwent CXL as described above. They were harvested twenty-four hours after CXL. First, the culture medium was discarded and the cells were trypsinized and washed with PBS. To demonstrate alpha-SMA, the cells were incubated with 0.5 mL PERM (permeabilization) solution for 10 minutes; then the cells were washed once with PBS followed by incubation with FITC-conjugated mouse monoclonal antibodies (IgG2a) against human alpha-SMA (100 *μ*g/10^4^ cells) for 30 minutes in the dark at room temperature. For CD34, a FITC-conjugated monoclonal antibody (IgG1) was used directly at a concentration of 200 *μ*g/mL followed by an incubation step for 30 minutes in the dark at room temperature. To prove the specificity of the staining, an isotype control experiment for each primary IgG-subtype antibody was performed. In the following steps, all cell preparations were washed twice with PBS and analyzed using a FACSCanto flow cytometer (BD Biosciences, Heidelberg, Germany) and the evaluation was performed with WinMDI software (Version 2.9).

### 2.8. Protein Measurement

After taking the supernatant for ELISA, the total protein concentration of each well was measured following detachment of the cells with 150 *μ*L CelLytic M (Sigma, Deisenhofen, Germany). Protein quantity was determined according to the method of Bradford [21], which is based on the formation of a complex between the dye brilliant blue G and proteins in solution. The absorbance was measured at 595 nm and the concentrations were quantified using bovine serum albumin (BSA) as a standard protein.

### 2.9. Measurement of Growth Factors and Interleukins

Five hours and 24 hours after CXL, the concentration of FGFb, HGF, TGF*β*1, VEGF, KGF, IL-1*β*, IL-6, and IL-8 in each well was measured by taking a 100 *μ*L aliquot of the supernatant of the wells. Measurements were performed by ELISA (KOMABIOTECH, Seoul, Korea) with the following measurement ranges.

The measurement ranges are as follows: FGFb, 1000-8 pg/mL; HGF, 8000-60 pg/mL; TGF *β*1, 2000-16 pg/mL; VEGF, 2000-16 pg/mL; KGF, 1000-8 pg/mL; IL-1*β*, 1000-8 pg/mL; IL-6, 600-10 pg/mL; and IL-8, 8000-60 pg/mL. Measured concentrations below the respective minimum values were considered as zero. The cytokine concentrations were quantified by using a human recombinant FGFb, HGF, TGF*β*1, VEGF, KGF, IL-1*β*, IL-6, and IL-8 as standard. The measurements were performed exactly following the manufacturers' ELISA-protocols. In each well, the concentration of the cytokines in the supernatant was standardized to the cell protein concentration of the respective well. The absorbance was measured at 450 nm (Model 550 Bio-Rad Laboratories GmbH, München, Germany). The experiments were repeated five times using keratocyte cultures from five different patients.

### 2.10. Statistical Analysis

For statistical analysis SPSS 16.0 was used. Data are represented as mean ± standard deviation (SD). Statistical analysis was performed using the Mann-Whitney *U* test. *P* values below 0.05 were considered statistically significant.

## 3. Results

### 3.1. KC Keratocyte Viability

**Figure 1:**
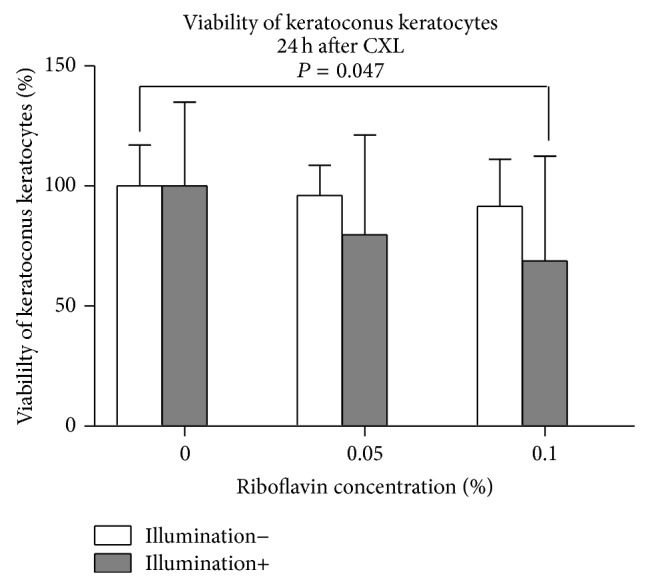
Viability of human KC keratocytes 24 hours following CXL. Application of 0.05% or 0.1% riboflavin-5-phosphate solution without illumination or the UVA-light illumination alone did not show a significant impact on keratocyte viability compared to controls (*P* > 0.34). Using 0.1% CXL, viability of KC keratocytes decreased significantly (*P* = 0.047).

Results of the Alamar Blue assay are shown in Figure 1 (*n* = 5). With the separate use of riboflavin or UVA-light illumination, viability of KC keratocytes did not change significantly compared to controls (*P* > 0.34). Following CXL, using 0.1% riboflavin, KC keratocyte viability was significantly decreased (*P* = 0.047) compared to controls.

### 3.2. KC Keratocyte Apoptosis

**Figure 2:**
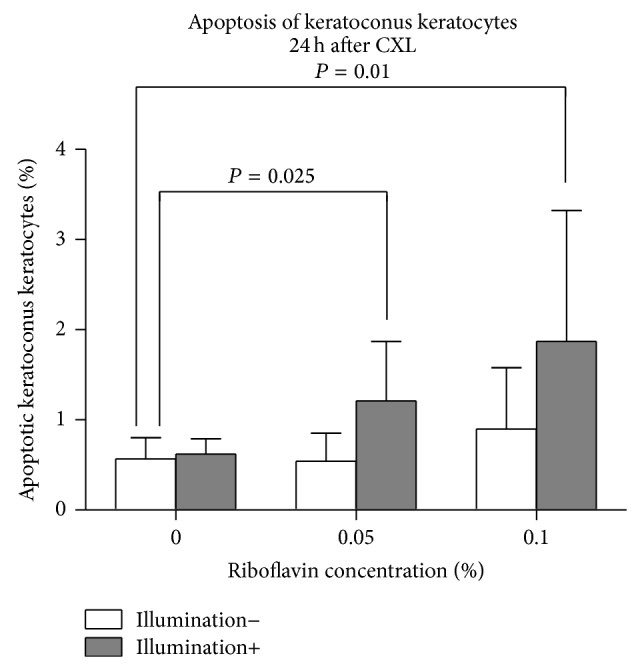
Percentage of apoptotic KC keratocytes 24 hours following CXL. The use of riboflavin or UVA-light illumination separately had no significant impact on the percentage of apoptotic keratocytes compared to controls (*P* > 0.262). Following CXL using 0.05% or 0.1% riboflavin, the percentage of apoptotic KC keratocytes was significantly increased (*P* = 0.025 and *P* = 0.01, resp.).

Figure 2 shows the percentage of apoptotic KC keratocytes 24 hours following CXL (*n* = 5). Using the Apo-Direct kit, there was no significant difference in the percentage of apoptotic KC keratocytes using riboflavin or UVA-light illumination separately (*P* > 0.26). However, using CXL with 0.05% or 0.1% riboflavin significantly increased the percentage of apoptotic KC keratocytes compared to controls (*P* = 0.025 and *P* = 0.01, resp.).

### 3.3. KC Keratocyte Proliferation

**Figure 3:**
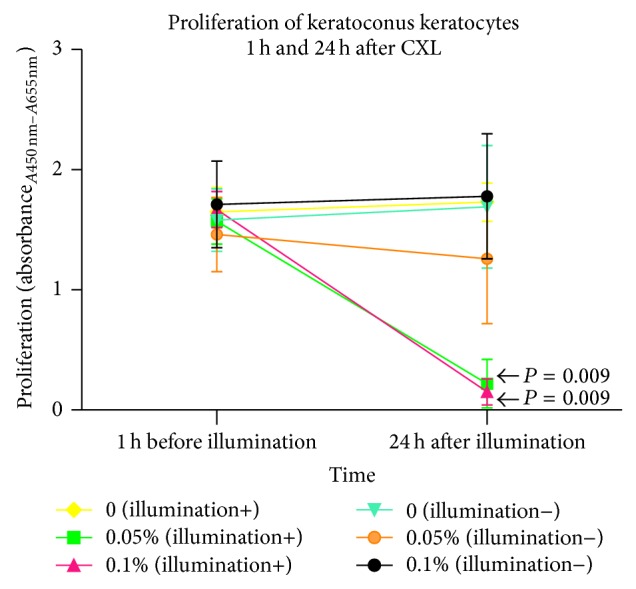
Proliferation of human KC keratocytes 24 hours after CXL. Twenty-four hours after illumination, the proliferation of keratocytes was significantly inhibited using 0.05% or 0.1% riboflavin (*P* = 0.009 for both concentrations) compared to controls. Proliferation of keratocytes remained unchanged using riboflavin or illumination alone (*P* > 0.25). The 1 h before illumination group was tested as a starting point in cell growth under the same conditions.

Proliferation of KC keratocytes is displayed in Figure 3 (*n* = 5). Using riboflavin or UVA-light illumination separately, proliferation of KC keratocytes did not change significantly (*P* > 0.25). Twenty-four hours after CXL, proliferation of KC keratocytes was inhibited significantly using 0.05% or 0.1% riboflavin concentrations (*P* = 0.009 for both concentrations), compared to controls.

### 3.4. CD34 Expression of KC Keratocytes

**Figure 4:**
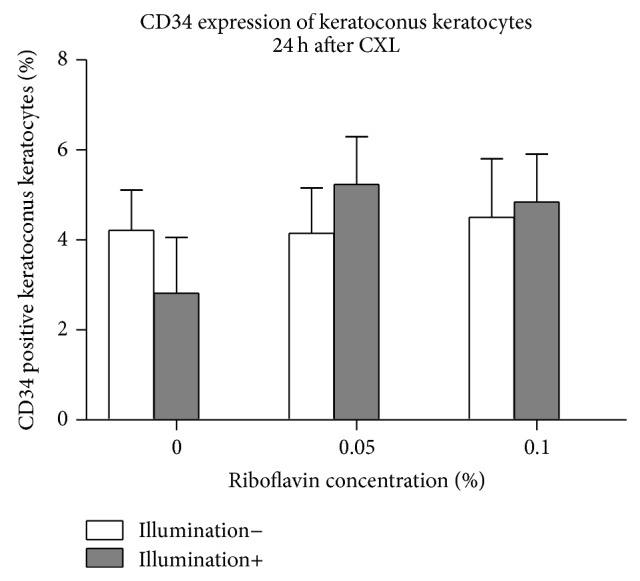
CD34 expression of keratoconus keratocytes 24 hours after CXL. Using riboflavin or illumination only, expression of CD34 in KC keratocytes did not change significantly compared to controls (*P* > 0.873). Twenty-four hours after CXL, CD34 expression of keratocytes also remained unchanged at both riboflavin concentrations (*P* = 0.15 and *P* = 0.34), compared to controls.

CD34 expression of KC keratocytes twenty-four hours following CXL is summarized in Figure 4 (*n* = 5). Using riboflavin or illumination separately or application of CXL resulted in no significant change in CD34 expression compared to controls (*P* > 0.15).

### 3.5. Alpha-SMA Expression of KC Keratocytes

**Figure 5:**
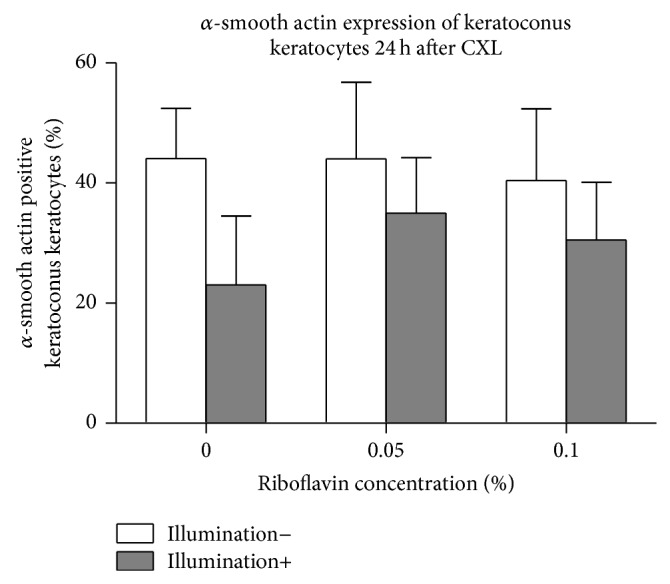
*α*-SMA expression of KC keratocytes 24 hours after CXL. Twenty-four hours following treatment, there was no significant difference in the percentage of alpha-SMA positive KC keratocytes using riboflavin or UVA-light illumination separately (*P* > 0.16). Using CXL, *α*-SMA expression in keratocytes also remained unchanged at both riboflavin concentrations (*P* = 0.15 and *P* = 0.06), compared to controls.

Alpha-SMA expression in KC keratocytes twenty-four hours following CXL is summarized in Figure 5 (*n* = 5). There was no significant difference in the percentage of alpha-SMA positive KC keratocytes using riboflavin or UVA-light illumination separately (*P* > 0.16). Using CXL, there was also no significant difference at 0.05% or 0.1% riboflavin concentrations (*P* = 0.15 and *P* = 0.06, resp.) in alpha-SMA expression of keratocytes compared to controls.

### 3.6. Growth Factor and Interleukin Secretion of KC Keratocytes

**Figure 6:**
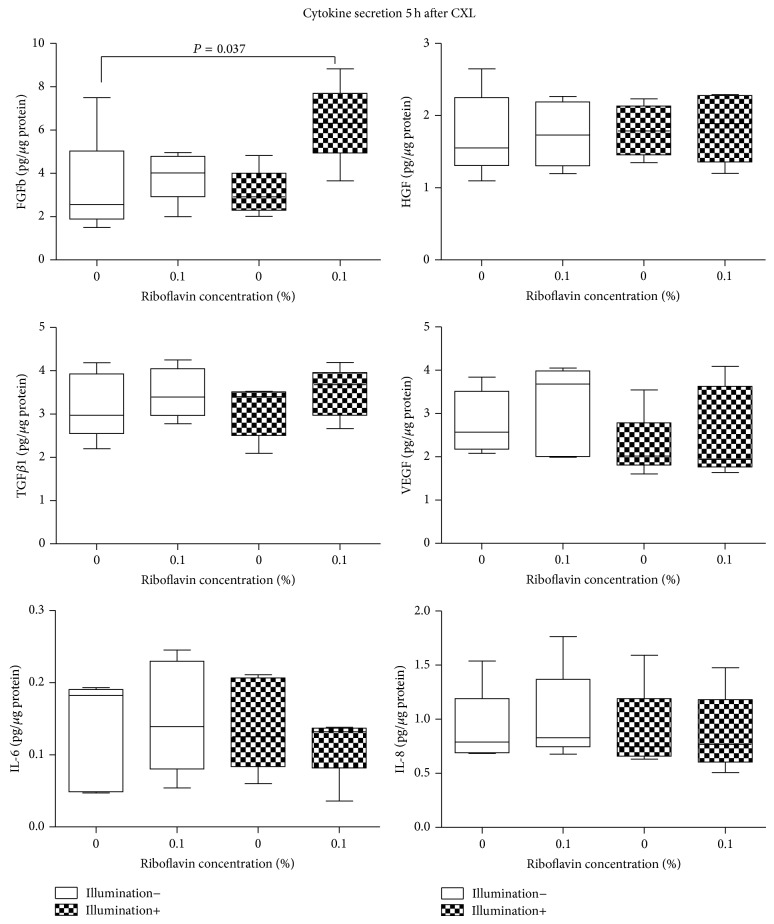
Cytokine secretion 5 hours after cross-linking. Significant differences are indicated.

FGFb, KGF, VEGF, HGF, TGF*β*1, IL-1*β*, IL-6, and IL-8 concentrations 5 h after CXL are summarized in Table 1 and Figure 6 (*n* = 5). The secretion of KGF and IL-1*β* was below the limit of detection in the treated and untreated cell cultures 5 h after treatment. With the separate use of riboflavin or UVA-light illumination, growth factor and interleukin secretion of KC keratocytes did not differ significantly from untreated controls (*P* > 0.34, *P* > 0.08).

Five hours after CXL, using 0.1% riboflavin and illumination, the mean FGFb concentration was 6.32 ± 1.84 pg/*μ*g protein in the supernatant of the medium of KC keratocytes. This was significantly higher than FGFb concentration in untreated KC keratocyte cultures (3.28 ± 2.40 pg/*μ*g protein; *P* = 0.037). We could not detect changes in the secretion of VEGF, HGF, TGF*β*1, IL-6, or IL-8 of KC keratocytes at any of the examined groups 5 h following CXL, compared to controls (*P* > 0.35).

**Figure 7:**
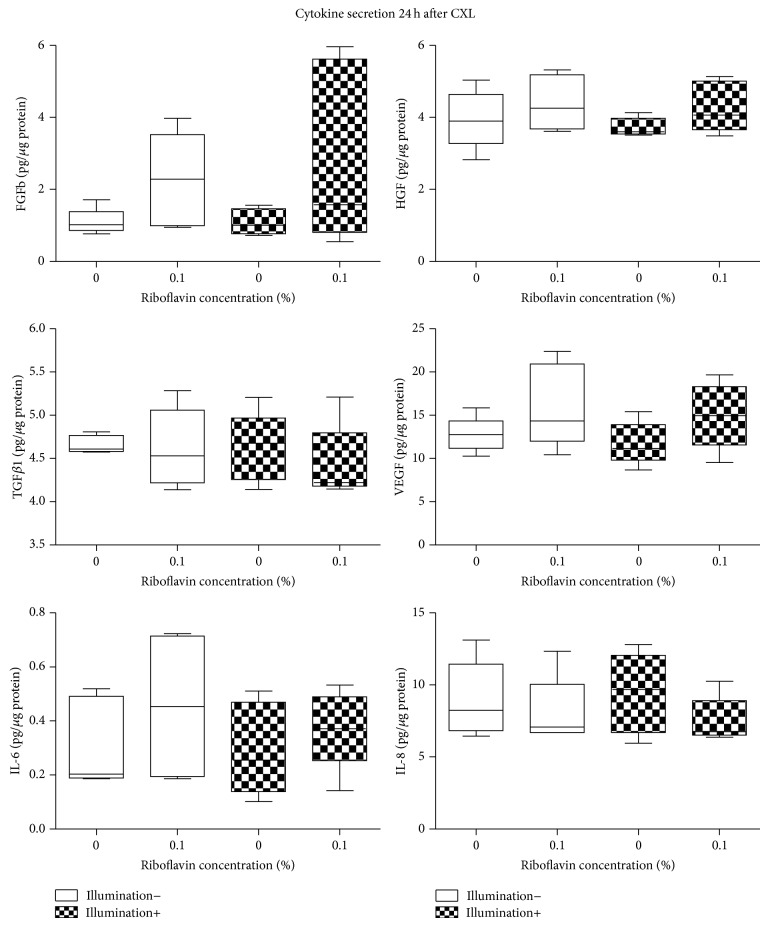
Cytokine secretion 24 hours after cross-linking. No significant difference could be detected compared to untreated controls.

Table 2 and Figure 7 display FGFb, KGF, VEGF, HGF, TGF*β*1, IL-1*β*, IL-6, and IL-8 concentrations 24 h after CXL (*n* = 5). KGF and IL-1*β* secretion was below the detection limit in the treated and untreated cell cultures 24 h after CXL. With the separate use of riboflavin or UVA-light illumination, growth factor and interleukin secretion of KC keratocytes did not change significantly compared to controls (*P* > 0.12, *P* > 0.35). Twenty-four hours after CXL, FGFb, VEGF, HGF, TGF*β*1, IL-6, and IL-8 secretion also did not change significantly compared to untreated control KC keratocyte cultures (*P* > 0.12).

## 4. Discussion

Corneal collagen cross-linking was first introduced by Wollensak et al. to inhibit or stop disease progression and to increase the biomechanical stability of the cornea in KC [4]. Cross-linking was confirmed to flatten keratometric readings, reduce cone progression, and even improve best corrected visual acuity [22, 23].

Although CXL is in clinical use, to the best of our knowledge the impact of CXL on KC keratocytes has not yet been analyzed in detail.

The present study determined that, in human KC keratocytes, viability decreases, apoptosis is triggered, and proliferation is inhibited; however multipotent haematopoietic stem cell transformation and myofibroblastic transformation remains unchanged 24 hours after treatment.

Viability and apoptosis of normal keratocytes have been analyzed by several authors following CXL in the past: Wollensak et al. have shown an abrupt cytotoxic effect* in vitro* on normal porcine keratocytes [22] and Grobe and Reichl detected significantly decreased cell viability in a human keratocyte cell line [23]. Recently, our research group has shown that CXL decreases viability; however this does not have an impact on apoptosis of primary normal human keratocytes* in vitro*. Interestingly, in primary human KC keratocytes our present study revealed the triggering of apoptosis 24 hours after CXL treatment.

Kim et al. and Kaldawy et al. noted that an increased percentage of apoptotic keratocytes is present in human KC corneas compared to normal human controls [24, 25]. Macé et al. [26] suggested that cell loss resulting from antiproliferative and hyperapoptotic phenotypes may be responsible for the pathogenesis of KC. In addition, changes in corneal protein pattern, increase in enzymatic activities, and cell apoptosis are also thought to be part of KC progression [27]. Chwa et al. [28] described an increased basal generation of reactive oxygen species and reactive nitrogen species in KC keratocytes.

Five to 30 months following CXL therapy, Messmer et al. [29] described keratocyte damage and increased antiapoptotic bax and/or antiapoptotic surviving protein expression in keratocytes compared to untreated KC controls. In our opinion, the programmed cell death of keratocytes has to be further analyzed at the cellular-subcellular level and alterations of the apoptotic pathways in normal and KC keratocytes have to be described (also following CXL), so that we are better able to understand the disease.

The decreased proliferation of KC keratocytes 24 hours after CXL in our present study is not in accordance with the published* in vivo* results six months after treatment, which have shown an increased proliferation (Ki67 positive) of the cells at this time point [30]. In our opinion the triggered proliferation at six months after CXL is part of the late wound healing response and proliferation after 24 hours is likely to be also inhibited* in vivo*. Our previous study also reported on decreased proliferation of primary human keratocytes 24 hours following chlorine e6 photodynamic therapy in accordance with our present CXL results [13].

CD34 positive keratocytes are confirmed to be multipotent hemopoietic stem cells which may play a role in cytoadhesion and signaling related to differentiation and proliferation [31–33]. Meanwhile, *α*-SMA is agreed to be a marker of myofibroblasts. Corneal myofibroblasts synthesize and secrete collagen I and play an important role in wound healing and contraction [32]. Furthermore, recent studies revealed that, by expressing toll-like receptor (TLR) [32], corneal myofibroblasts may take part in pathogen clearance. It is also suggested that myofibroblast differentiation downregulates keratocyte CD34 expression [23].

Several authors reported on the occurrence of early bacterial or acanthamoeba keratitis after CXL. Our previous study found induced myofibroblastic transformation and multipotent haematopoietic stem cell transformation in normal human keratocytes 24 hours after CXL [33]. These results do not support the hypothesis that CXL suppresses the inflammatory response of keratocytes, which leads to an increased incidence of infectious keratitis after CXL. In contrast, the inflammatory response should be promoted through CXL.

Most interestingly, in human KC keratocytes, CD34 and *α*-SMA expression remained unchanged following CXL. In other words, the percentage of multipotent hemopoietic stem cells and myofibroblasts remained unchanged after treatment, compared to untreated KC keratocytes. Therefore, we also do not see an increased risk of infectious keratitis in KC patients after CXL. At least this should not be correlated with intraoperative modification of the cellular response.

In a previous study, using chlorine e6 as a photosensitizer for photodynamic therapy, there were also no significant changes in CD34 expression of normal keratocytes due to this treatment; however, *α*-SMA expression decreased [33]. The reason for the difference in both studies could be explained by the use of KC keratocytes in our present study and the use of normal keratocytes in the previous study. However, the molecular pathways, which result in changes of CD34 and *α*-SMA expression in both keratocyte types, should be further analysed in the future.

In the corneal wound healing response cytokine-mediated interactions between epithelial cells, keratocytes, corneal nerves, lacrimal gland, tear film, and cells of the immune system are involved [34]. The present study determined that CXL triggers FGFb secretion of keratocytes transiently (5 hours). In the short term, CXL does not have an impact on HGF, TGF*β*1, VEGF, KGF, IL-1*β*, IL-6, and IL-8 secretion of KC keratocytes,* in vitro*.

FGF promotes angiogenesis and cell proliferation and migration and also induces the differentiation of keratocytes into a fibroblastic phenotype [35]. In accordance with this there was no impact of CXL on myofibroblastic transformation and multipotent hemopoietic stem cell transformation of KC keratocytes (Figures 4 and 5). In contrast, with decreased FGFb secretion after CXL in normal keratocytes, both (normal and KC) keratocyte forms were activated after treatment. Ley et al. described that ultraviolet light induces FGFb, due to DNA damage [36]. In accordance with this we saw an increased FGFb secretion after CXL and also induced apoptosis of KC keratocytes.

It is known that HGF and TGF*β*1 induce myofibroblastic transformation of keratocytes. Our present results with stable HGF and TGF*β*1 secretion of the cells 5 and 24 hours after treatment (Figures 6 and 7) were also in accordance with the myofibroblastic transformation or multipotent hemopoietic stem cell transformation results shown in Figures 4 and 5.

HGF and KGF are secreted through the stromal cells of the cornea and are known to inhibit the process of epithelialization [37–39]. With unchanged HGF and KGF secretion of KC keratocytes following CXL, we do not expect an impact of CXL on corneal epithelial proliferation or migration. Interestingly, in our previous work on normal keratocytes after CXL, both HGF and KGF were downregulated. The response of different keratocyte cell types on CXL should be further analyzed [16, 17].

Wilson et al. reported on the inhibitory effect of HGF on apoptosis of the cells. We demonstrated that, in normal keratocytes, higher concentration of HGF (compared to our present study) after CXL prevented keratocytes from apoptosis [38]. In contrast, in our present study, a relative lower HGF secretion of KC keratocytes might be related to triggered apoptosis [17].

Interestingly, in our recent study, 5 hours after CXL of normal keratocytes, VEGF secretion was significantly decreased, which indicates that in normal keratocytes CXL may inhibit hemangiogenesis and lymphangiogenesis in the short term [17]. In contrast, in our present study we verified no changes in VEGF secretion of KC keratocytes following CXL. Therefore, CXL may not have an impact on corneal hemangiogenesis and lymphangiogenesis in KC patients in the short term.

Bacon et al., Hirano, and Kick et al. reported on IL-1*β*, IL-6, and IL-8 as the key cytokines in corneal inflammation [40–42]. These cytokines were shown to be chemotactic for riboflavin in infected corneal cells, which can cause corneal destruction. Previously we detected significantly increased IL-6 secretion in normal keratocytes after CXL; however in our present study on KC keratocytes, IL-6 and IL-8 secretion did not change significantly after treatment. Therefore, we do not expect CXL to have an impact on the inflammatory response of KC patients, but we do expect a positive effect of the treatment (chemotactic for riboflavin) in nonkeratoconic corneas, for example, with infectious keratitis.

IL-1 is a key factor in the corneal wound healing cascade, which leads to keratocyte apoptosis and/or necrosis after epithelial injury [34, 43] and the effect appears to be mediated via the Fas/Fas ligand system [44]. In the present research, IL-1 was not detected in the supernatant of KC keratocytes, but it was present in normal keratocyte cultures [16]. This indicates that IL-1 does not seem to play a key role in the KC wound healing cascade after CXL, at least* in vitro*.

Oleinick and Evans have reported that IL-6-induced apoptosis is an important mode of photodynamic therapy induced cell death [45]. Furthermore, in research with IL-6 transfected cells, it was suggested that apoptotic response occurs by induction of IL-6 expression which activates bax and bcl-2 after photodynamic therapy [46]. In our present study, IL-6 remained unchanged after CXL in KC keratocytes; however, it was downregulated in normal keratocytes in a previous study [16].

In summary, cross-linking decreases viability, triggers apoptosis, and inhibits proliferation; however it does not have an impact on multipotent haematopoietic stem cell transformation and myofibroblastic transformation of human KC keratocytes* in vitro*. Thus, we oppose the idea that CXL may induce bacterial or acanthamoeba keratitis. CXL triggers FGFb secretion of KC keratocytes transiently (5 hours), which normalizes after 24 hours. CXL does not seem to have an impact on HGF, TGF*β*1, VEGF, KGF, IL-1*β*, IL-6, and IL-8 secretion of KC keratocytes in the short term. Alterations of intracellular pathways following CXL should be further analyzed in the future.

## Figures and Tables

**Table 1:** Concentration (pg/*μ*g protein) of different cytokines in keratoconus keratocyte cultures five hours after treatment.

	Keratocytes	Keratocytes + riboflavin	Keratocytes + 370 nm	Keratocytes + riboflavin + 370 nm	^*^ *P* value	^**^ *P* value	^***^ *P* value
FGFb	3.28 ± 2.40	3.89 ± 1.15	3.10 ± 1.06	6.32 ± 1.84	0.34	0.6	***0.037***
HGF	1.73 ± 0.58	1.74 ± 0.45	1.79 ± 0.36	1.83 ± 0.48	0.92	0.6	0.75
TGF*β*1	3.19 ± 0.76	3.48 ± .58	3.09 ± 0.61	3.51 ± 0.57	0.47	0.75	0.35
VEGF	2.79 ± 0.07	3.13 ± 1.04	2.24 ± 0.75	2.55 ± 1.05	0.75	0.08	0.35
KGF	n.d.	n.d.	n.d.	n.d.	n/a	n/a	n/a
IL-1*β*	n.d.	n.d.	n.d.	n.d.	n/a	n/a	n/a
IL-6	0.13 ± 0.08	0.15 ± 0.08	0.14 ± 0.06	0.11 ± 0.04	0.47	0.47	0.35
IL-8	0.91 ± 0.36	1.01 ± 0.34	0.89 ± 0.4	0.87 ± 0.37	0.6	0.47	0.92

Values indicate mean ± SD.

^*^ *P* values indicate the difference between “keratocytes” versus “keratocytes + 370 nm” groups (Mann-Whitney *U* test).

^**^ *P* values indicate the difference between “keratocytes” versus “keratocytes + riboflavin” groups (Mann-Whitney *U* test).

^***^ *P* values indicate the difference between “keratocytes” versus “keratocytes + riboflavin + 370 nm” groups (Mann-Whitney *U* test).

Significant values are shown in bold.

n.d. = not detectable.

n/a = not applicable.

**Table 2:** Concentration (pg/*μ*g protein) of different cytokines in keratoconus keratocyte cultures twenty-four hours after treatment.

	Keratocytes	Keratocytes + riboflavin	Keratocytes + 370 nm	Keratocytes + riboflavin + 370 nm	^*^ *P* value	^**^ *P* value	^***^ *P* value
FGFb	1.1 ± 0.36	2.26 ± 1.3	1.09 ± 0.36	2.88 ± 2.53	0.12	0.75	0.3
HGF	3.94 ± 0.8	4.39 ± 0.77	3.72 ± 0.26	4.28 ± 0.71	0.35	0.35	0.6
TGF*β*1	4.66 ± 0.1	4.62 ± 0.46	4.62 ± 0.4	4.43 ± 0.44	0.6	0.92	0.12
VEGF	12.75 ± 2.01	16.02 ± 4.82	11.7 ± 2.47	14.92 ± 3.8	0.18	0.35	0.25
KGF	n.d.	n.d.	n.d.	n.d.	n/a	n/a	n/a
IL-1*β*	n.d.	n.d.	n.d.	n.d.	n/a	n/a	n/a
IL-6	0.31 ± 0.16	0.45 ± 0.26	0.29 ± 0.17	0.37 ± 0.15	0.53	0.6	0.75
IL-8	8.95 ± 2.64	8.11 ± 2.41	9.43 ± 2.79	7.52 ± 1.59	0.47	0.92	0.35

Values indicate mean ± SD.

^*^ *P* values indicate the difference between “keratocytes” versus “keratocytes + 370 nm” groups (Mann-Whitney *U* test).

^**^ *P* values indicate the difference between “keratocytes” versus “keratocytes + riboflavin” groups (Mann-Whitney *U* test).

^***^ *P* values indicate the difference between “keratocytes” versus “keratocytes + riboflavin + 370 nm” groups (Mann-Whitney *U* test).

n.d. = not detectable.

n/a = not applicable.
